# Pepper Mottle Virus and Its Host Interactions: Current State of Knowledge

**DOI:** 10.3390/v13101930

**Published:** 2021-09-25

**Authors:** Miao Fang, Jisuk Yu, Kook-Hyung Kim

**Affiliations:** 1 Department of Agricultural Biotechnology, Seoul National University, Seoul 08826, Korea; fmmy0506@gmail.com; 2 Plant Genomics and Breeding Institute, Seoul National University, Seoul 08826, Korea; mago03@snu.ac.kr; 3 Research Institute of Agriculture and Life Sciences, Seoul National University, Seoul 08826, Korea

**Keywords:** pepper mottle virus, *Potyvirus*, pepper resistance gene, virus–host interaction

## Abstract

*Pepper mottle virus* (PepMoV) is a destructive pathogen that infects various solanaceous plants, including pepper, bell pepper, potato, and tomato. In this review, we summarize what is known about the molecular characteristics of PepMoV and its interactions with host plants. Comparisons of symptom variations caused by PepMoV isolates in plant hosts indicates a possible relationship between symptom development and genetic variation. Researchers have investigated the PepMoV–plant pathosystem to identify effective and durable genes that confer resistance to the pathogen. As a result, several recessive *pvr* or dominant *Pvr* resistance genes that confer resistance to PepMoV in pepper have been characterized. On the other hand, the molecular mechanisms underlying the interaction between these resistance genes and PepMoV-encoded genes remain largely unknown. Our understanding of the molecular interactions between PepMoV and host plants should be increased by reverse genetic approaches and comprehensive transcriptomic analyses of both the virus and the host genes.

## 1. Introduction

*Pepper mottle virus* (PepMoV), which is in the genus *Potyvirus* and the family *Potyviridae* [1], has been isolated from economically important solanaceous plants including pepper (*Capsicum* sp.), potato, and tomato in North America, India, and Asia [2,3,4,5,6,7,8,9]. PepMoV is transmitted by several species of aphids but can also spread via mechanical inoculation, grafting, and infected seeds [10,11]. PepMoV causes various symptoms in host plants, including severe or mild mottling mosaic, necrosis, vein clearing or necrosis, and leaf curling or yellowing [12,13]. When pepper plants are co-infected with PepMoV and cucumber mosaic virus, synergistic disease development is the result [14,15].

PepMoV has a single-stranded positive-sense RNA genome that is about 10 kb in length [13,16,17]. The PepMoV RNA is expressed as a large polyprotein, which is catalyzed and cleaved into smaller mature proteins in the host [17,18]. Variation in symptoms, pathogenicity, and molecular properties among PepMoV isolates in Korea suggested that certain virus-encoded proteins determine host specificity or pathogenicity [4,19]. Recent studies of compatible/incompatible responses between PepMoV and host plants revealed dynamic interactions between PepMoV-encoded viral proteins and host proteins [20,21,22]. The development of full-length infectious clones of PepMoV has enabled researchers to investigate the interaction between PepMoV and hosts as well as to explore the feasibility of using PepMoV as a viral vector for the stable expression of heterologous genes in plants [23,24]. Here, we review what is know about PepMoV with a focus on recent major findings concerning the interactions between PepMoV and its plant hosts.

## 2. Diversity and Pathogenicity

PepMoV was reported first in Arizona and Florida in the early 1970′s and is considered as a species in the *Potyvirus* genus (lineage 5; [25,26]). Compared to potato virus Y (PVY), which is one of the major potyviruses infecting pepper, PepMoV forms relatively long and thin pinwheel inclusions in infected leaves and also differs in the symptoms it induces, its serological characteristics, the molecular weights of its proteins, and its nucleic acid contents [12,27,28].

To date, PepMoV has been reported from many regions in North America, East Asia, and India, and full genome sequences of 23 isolates are available and sequences of 45 isolates are partially released at the NCBI database. A phylogenetic analysis based on the deduced amino acid sequences indicated that all 13 Korean isolates of PepMoV formed one cluster that was distinct from American isolates [4]. A recent report indicated that full genome sequences of an isolate of PepMoV from Hunan China (PepMoV HN) are closely related to 18 PepMoV isolates from Korea that reported in previous studies [4,29,30]. However, when the authors of the latter study analyzed coat protein (*CP*) genes of nine PepMoV isolates from pepper in Southern China, they detected two distinct groups and subgroups.

Based on its genetic variation and on its symptom severity and pathogenicity on different host plants, researchers divided 13 Korean isolates of PepMoV into two groups [4,13]. Having acquired data on the highest ratio of synonymous (dS) to non-synonymous (dN) base substitutions for the P1 and 6K2 genes of PepMoV and on amino acid (aa) variation encoded by the 6K2 gene, Kim et al. suggested that the P1 and 6K2 genes might be involved in PepMoV host specificity and pathogenicity [4]. As discussed later in this review, Kim et al. also described a system that could be used to identify viral-encoded proteins affecting pathogenicity and host specificity.

## 3. Genome Organization

**Figure 1:**
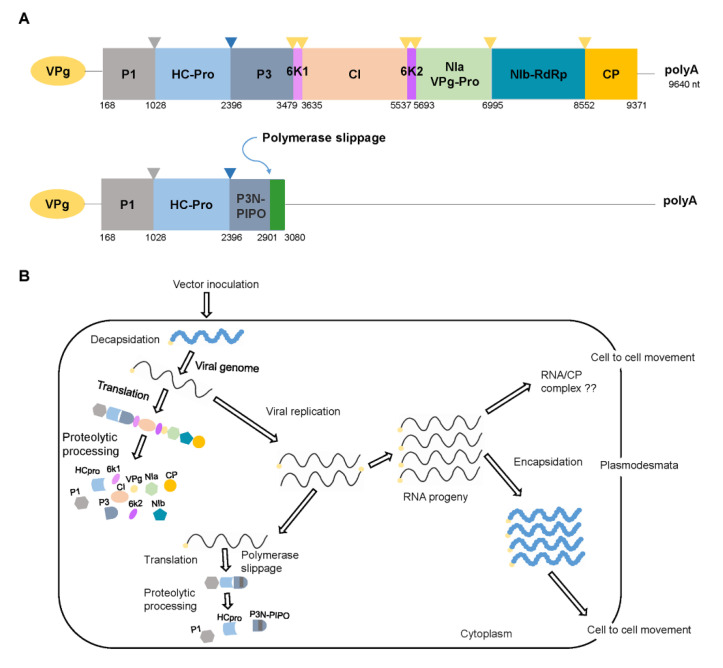
Genome organization and schematic representation of replication of pepper mottle virus (PepMoV). (**A**) The genomic maps of PepMoV. The genome is translated into a large polyprotein that is catalyzed by three potyviral proteases (P1, HC-Pro, and NIa) and cleaved into 10 mature viral proteins (marked in different colors). The next depicted represents a short open reading frame, termed P3N-PIPO, embedded within the P3-encoding region of the polyprotein. (**B**) Schematic representation of replication in a plant cell. The cycle begins (left upper corner) when the viral particle or RNA enters the cell from infected cells or initially inoculated by its vector. The genomic RNA undergoes decapsidation, translation, and proteolytic processing to generate mature proteins. The replication complex uses the positive genomic RNA to generate a complementary negative genomic RNA, which functions as a template for the synthesis of numerous genomic RNAs. After replication, the progeny RNAs can be encapsidated and acquired by vectors to be transmitted again, or they can move to adjacent cells through plasmodesmata.

Like all potyviruses, PepMoV forms a flexuous rod-shaped virion that consists of about 2000 copies of CP subunits with an Mr of 30.8 kDa [31]. The virion contains a positive-sense single-stranded viral genomic RNA with a genome-linked protein (VPg) at its 5′-terminal end and a poly(A) tail at its 3′-terminal end [17,31]. The PepMoV genome is translated into a large polyprotein that is catalyzed by three potyvirus proteases (P1, HC-Pro, and NIa) and catalyzed into 10 mature viral proteins (P1, HC-Pro, P3, 6K1, CI, 6K2, VPg, NIa, NIb, and CP) [16,17]. The existence of a short open reading frame, termed P3N-PIPO, embedded within the P3-encoding region of the polyprotein is a universally conserved feature and has conserved coding capacity throughout the genus [32,33,34] (Figure 1A).

Both of the approaches described in the previous paragraph have been used to construct full-length cDNA clones of PepMoV. A Korean isolate of PepMoV (PepMoV-Vb1) was cloned downstream of a bacteriophage SP6 promoter and was tagged with green fluorescent protein (GFP), which was inserted between the coding regions for NIb and CP in the plasmid [29]. In vitro RNA transcripts from this clone were infectious and stably expressed GFP in tobacco and pepper plants [29]. Although infectious clones are useful and fundamental tools in studying virus–host interactions, some challenges remain in constructing and stably delivering infectious clones into host plant using *E. coli*-based plasmids. For example, toxicity to *E. coli* has been reported for plasmids containing full-length clones of several viruses including citrus tristeza virus, tobacco etch virus (TEV), and influenza A virus; such toxicity makes it difficult or even impossible to use the clone for molecular manipulation [35,36,37]. During their modification and construction, full-length clones in *E. coli* have also been reported to be unstable for plant RNA viruses including potyviruses [36,38,39,40,41] and tobraviruses [42,43]. To reduce or minimize undesired toxic effects or instability in *E. coli*, insertion of introns into the viral genome has been extensively used [38,40]. For example, insertion of the potato ST-LS1 intron 2 sequence into the NIa coding region of PepMoV increased the stability of the infectious clone [23]. Relative to traditional pPepMoV infectious clones, the modified infectious clone inserted plant intron into pPepMoV could restore infectivity and maintain plasmid stability. *Agrobacterium*-mediated inoculation of this modification clone showed faster symptom induction compared to non-modified clones when the same amount of *Agrobacterium* cell suspension was inoculated in plants [23]. In addition, the resulting symptom intensity was similar to that following sap inoculation [23].

## 4. Replication and Movement: Functions of Viral Proteins

In research on the role of virus-encoded proteins during the virus infection process, use of the infectious full-length cDNA clones has provided reliable information on viral RNA replication and movement [44,45]. Almost all of the potyviral proteins are involved in viral replication [31] (Figure 1B). For example, the potyviral proteins HC-Pro, CI, VPg, NIb, and CP have multiple functions during viral infection, and CI, CP, HC-Pro, VPg, and P3N-PIPO have been implicated in viral intercellular movement [44]. The protein functions of PepMoV are largely unknown. However, the possible roles of PepMoV-encoded proteins could be expected from the reported functions of the other closely related potyviruses.

The P1 protein of potyviruses is a chymotrypsin-like serine proteinase that cleaves itself at C-terminus [31,46]. P1 is the most divergent and variable protein among potyvirus-encoded proteins [46,47]. The TEV P1 protein has been shown to function *in trans* to stimulate genome amplification [48]. The function of clover yellow vein virus P1 has been reported for its involvement in eIF4E-mediated recessive resistance [49]. The potato virus V (PVV) P1 does not have direct association with RNA silencing suppression, but self-cleavage activity of P1 affects RNA silencing suppression indirectly by modulating function of HC-Pro [50].

The HC-Pro is a cysteine protease and well-established multitasking protein that is involved in many potyviral infection processes such as aphid-mediated transmission, RNA silencing suppression, genome replication, symptom expression, and long-distance movement [51,52]. HC-Pro could interact with several other potyviral proteins and many host factors [47]. Two conserved motifs, i.e., the N-terminal ‘KITC’ and the C-terminal ‘PTK’ motifs, have been identified in HC-Pro [53]. Site-directed mutation replacing lysine to glutamic acid (K59E) within the KITC motif using several PVY isolates abolishes the interaction of HC-Pro with aphid stylets and aphid transmissibility of PVY [53]. It is reported that the PVY HC-Pro interacts with three *Arabidopsis* 20S proteasome subunits (PAA, PBB, and PBE), which is related to the antiviral response [54]. In addition, HC-Pro from three potyviruses, including potato virus A (PVA), PVY, and TEV could interact with the eukaryotic translation initiation factors (eIF4E) and eIF(iso)4E of *Nicotiana tabacum* and eIF(iso)4E and eIF4E of potato suggesting possible new role(s) in potyvirus infection cycle [55].

The protein P3 is also one of the well-characterized multifunctional potyviral proteins. Dual roles of TEV P3 in virus movement and replication have been reported [56,57]. A polymerase slippage mechanism on P3 cistron leads to the production of P3N-PIPO [32], which localizes at PD and involves in the viral cell-to-cell movement in conjunction with CI protein [58]. P3 plays crucial roles as virulence and symptom determinants [59].

The 6K1 of plum pox virus (PPV) is required for viral replication and is a necessary viral element of the viral replication complexes (VRC) at the early infection stage [60].

The multifunctional CI protein, as part of the VRC, participates in viral genome replication. In addition to replication, it also functions in viral cell-to-cell and long-distance systemic movement, probably by interacting with the recently reported viral P3N-PIPO protein [44,58,61]. There is genetic evidence suggested that CI protein of TEV interacts directly with plasmodesmata and CP-containing ribonucleoprotein complexes to facilitate intracellular movement [62]. The lettuce mosaic virus CI has been shown to interact with the viral VPg and with lettuce eIF4E [63] and involved in the eIF4E-mediated resistance-breaking [64,65].

The potyviral 6K2 protein has been found to be involved in long-distance movement and symptom development [66,67,68]. In addition, potyviral 6K2 exhibits critical components of the VRC with NIb, HC-Pro, P3, CI, and NIa [69]. The potyviral 6K2 induces proliferation of ER membrane for construction of VRCs at ER exit sites in cellular coatomer protein I- and II-dependent manner [67,70]. The TEV 6-kDa protein is membrane associated and has been shown to be necessary for virus replication [71]. It contains transmembrane (TM) domain at N-terminal region and putatively luminal domain at C-terminal region [72,73]. The TM domain of potyviral 6K2 protein is typically required for targeting and anchoring to the ER membrane [72]. The N-terminal region of TEV 6K2 includes a D(X)E motif which is crucial for ER exit of the 6K2-induced replication vesicles [73].

Potyvirus VPg contains two nuclear localization signals (NLSs) and nucleotide triphosphate binding motifs [74]. VPg is required for several viral processes, including translational initiation of viral RNA and replication [74]. Potyviral VPg can form an intrinsically disordered state of the protein and this structural flexibility provides accessible interaction complexes with different virus or host proteins to enable its diverse functions [47,74]. Including PVA VPg, it requires host eIF4Es to promote viral RNA replication as well as the viral translation products [75]. In contrast, interaction between VPg and host eIF4E and eIF(iso)4E might be involved in translation inhibition in host cellular mRNAs [75,76]. VPgs in PVY, TEV, and turnip mosaic virus (TuMV) can inhibit cellular cap-dependent translational initiation in vitro through binding with eIF4E or eIF(iso)4E [77,78,79]. The interaction between VPg and eIF4E is also related to recessive resistance response against several potyviruses [80,81]. TuMV 6K2–VPg–NIa complex, membrane-associated precursor form, is found within vesicular structures derived from the ER where replication might occur [82]. TuMV VPg also has RNA silencing suppressor activity by inducing degradation of suppressor of gene silencing 3 (SGS3), which is involved in RNA silencing pathway [83].

Potyvirus NIa-Pro is a cysteine protease and generally functions proteolytic processing of the potyviral polyprotein [47]. NIa-Pro accesses differential cleavage efficiency, and it affects host range and viability of potyviruses [84]. Previous studies have described how PVY and PepMoV NIa were able to elicit *Ry*-mediated HR in *Solanum stoloniferum* by sharing the same recognition/cleavage site for NIa [85]. Expression of NIa-Pro interferes ethylene signaling pathway and enhances aphid fecundity in TuMV-infected *Arabidopsis* [86]. NIa-Pro relocalization from cytoplasm and nucleus to the vacuole of the cell during TuMV and PVY infections when in the presence of the aphid vector has reported [87]. This relocalization confers the ability to promote vector performance to potyvirus NIa-Pro [87]. Recent study suggested that PepMoV NIa was involved in pathogenicity and suppression of host antiviral defense response [88].

NIb of potyviruses acts as an RNA-dependent RNA polymerase (RdRp) or RNA replicase, and is therefore required for potyviral genome replication [69]. Beyond its major role as an RdRp during viral infection, NIb also has additional functions such as a recruiter that interacts with many pro-viral host factors participated in the assembly and activation of the VRC, a suppressor of host defense response, a target of host antiviral defense, and an elicitor that activates effector-triggered immunity (ETI) [69]. One of the characterized functions of PepMoV NIb was associated with *Pvr4*- or *Pvr9*-mediated hypersensitive response (HR) [20,21].

The potyviral CP has also been reported to participate in the regulation of viral RNA replication [31,89]. Potyvirus CP is indispensable for viral intra- and intercellular movement [90,91]. Including PVA CP, the CP-vRNA interaction regulates virion assembly/disassembly and coordinates switch between viral RNA translation and replication [47,92]. Both terminal regions of the PVY CP have a crucial role in PVY infectivity. However, only the N-terminal region of CP is essential for virus-like particle (VLP) formation [89]. Recent findings for TuMV CP also suggest functions of the N-terminal region of CP in virion maturation and/or termination of virion formation. However, several studies demonstrated that the involvement of the N-terminal of potyviral CP in cell-to-cell movement and systemic infection varies from virus-to-virus [93]. N-terminal domains of TuMV and zucchini yellow mosaic virus CPs were dispensable for viral cell-to-cell and long-distance movements. However, the same regions in TEV and PVY are necessary for establishing cell-to-cell movement and systemic infection [89,93,94,95]. In contrast, C-terminal regions of TuMV, PVY, and TEV CPs were shown to be associated with viral cell-to-cell and long-distance movement [90,92,94]. The aromatic residue tryptophan at core domain (W^122^) of CP in tobacco vein banding mosaic virus, which is highly conserved residue among potyviruses, plays a role in maintaining stability of CP during viral replication and this involvement in viral cell-to-cell movement was also observed with the same residue of watermelon mosaic virus and PVY [96]. An additional role as the pathogenicity determinant for CP has been reported in PVY [97]. The highly conserved DAG motif in the N-terminal domain of CP is responsible for aphid transmission by mediating the interaction between CP and HC-Pro [98,99].

So far, extensive research has mainly focused on the defense response-related PepMoV-encoded proteins and their corresponding host genes. Although the functions of HC-Pro remain to be established in PepMoV, the protein is likely to be involved in replication and systemic movement. Likewise, PepMoV NIb might have roles in symptom development and virus multiplication, which is supported by our recent study (unpublished data).

## 5. Resistance Genes against PepMoV

Plants have evolved multi-layered systems to defend against viral invasion, including RNA silencing, regulation of RNA stability, ubiquitination-mediated protein degradation, autophagy, HR, *R* gene-mediated resistance responses, and systemic acquired resistance [47,100,101]. Several studies have described incompatible interactions between PepMoV and pepper plants; the host symptoms associated with those resistance responses have been used to identify efficient resistance (*R*) genes for application in plant breeding [20,102]. Characterized host genes are listed in Table 1.

### 5.1. Recessive Resistance Genes

**Figure 2:**
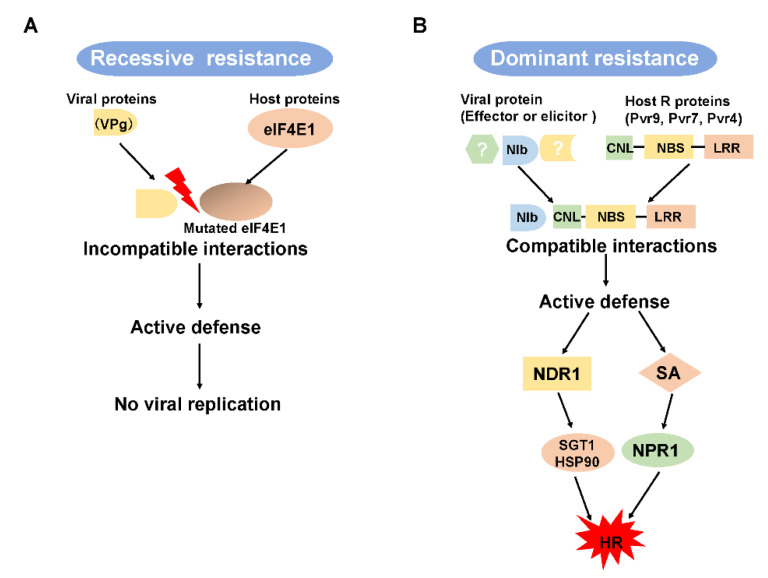
A scheme of host genes that may mediate recessive and dominant resistance and an explanation of the R protein-mediated signaling pathway. (**A**) Recessive resistance results from a host factor, the loss or mutation of which causes an incompatible interaction between a viral protein and a host protein. (**B**) Dominant resistance results from a compatible interaction between a viral effector and plant R proteins. Pvr9-mediated hypersensitive response requires several proteins, like NDR1 and the SGT1-HSP90 complex. Pvr9-mediated HR might also involve the SA pathway. NBS, nucleotide-binding site; LRR, leucine-rich repeat; CC, coiled-coil motif; HR, hypersensitive response; SA, salicylic acid.

In incompatible interactions between a plant virus and host, resistance responses can be mediated by recessive or dominant host genes (Figure 2) [21,80,81,106]. Recessive resistance genes are produced by the loss or mutation of a host factor that has an important function in disease development [15,100,107]. Recessive resistance genes are thought to be more durable and to provide more broad-spectrum resistance than dominant *R* genes [108]. Recessive resistance genes are more common than dominant resistance genes, especially against potyvirus infections, and usually function at the single-cell level and thereby limit cell-to-cell movement [81].

In *Capsicum* spp., *pvr1* and *pvr3* have been characterized as two unlinked recessive loci that confer distinct kinds of resistance to PepMoV [104]. The *pvr1* gene, which was identified in *C. chinense* PI159236 and PI152225, confers relatively broad resistance to PepMoV, TEV, and PVY [22,81]. In contrast, the *pvr3* gene, which was identified in *C. annuum* ‘Avelar’, confers a different type of resistance to PepMoV than to TEV and PVY [104]. The mechanisms of resistance responses in *Capsicum* spp. against PepMoV differ depending on whether the response is *pvr1-* or *pvr3*-mediated [15,104]. *C. chinense* PI 152225 and PI 159236, which contain *pvr1,* do not support replication of PepMoV at the cellular level, whereas *C. annuum* ‘Avelar’, which contains *pvr3* allows for PepMoV accumulation in inoculated leaves and its movement into the vascular system but not its spread into upper leaves [15,104,109]. However, this restriction of systemic movement was collapsed when PepMoV was co-infected with cucumber mosaic virus [15,109]. Other recessive genes, i.e., *pvr2* and *pvr6*, that are located on the pepper chromosomes 4 and 3, respectively, confer digenic recessive resistance to another pepper potyvirus, pepper veinal mottle virus [110]. However, the effect of *pvr2* and *pvr6* in response to the infection of PepMoV remains to be determined.

The eukaryotic translation initiation factors (eIF4Es) have been identified and cloned from diverse hosts as resistance genes that are natural, recessive, and inherited (Figure 2A) [80,111]. The *pvr1* locus encodes an eIF4E homolog, and *pvr6* is expected to encode eIF(iso)4E [81]. Recessive resistance against several potyviruses in plant hosts is conditioned by mutations in eIF4E and its isoforms [80,106,112]. Using transgenic tomato progeny with ectopic expression of the *pvr1*, researchers documented dominant resistance to several potyviruses, including PepMoV and TEV [113]. Moreover, resistance induced by mutation of eIF4E1 in tomato, obtained by TILLING platform or by Clustered Regularly Interspaced Palindromic Repeats/CRISPR-associated protein 9 (CRISPR/Cas9)-mediated targeted mutagenesis, was enhanced against PepMoV but not against TEV [22,114]. These studies indicated that *pvr1* or eIF4E greatly affects the resistance/susceptibility to plant viruses, especially to PepMoV, although the specific mechanism is unclear. In general, previous studies have suggested that eIF4E is also required for cell-to-cell movement and viral RNA replication during potyvirus infections [80,81,115]. The interaction between eIF4E and potyviruses will be discussed later in this review.

### 5.2. Dominant R Genes

Dominant *R* genes, corresponding to pathogen effector-encoding or avirulence (*Avr*) genes, confer an active resistance resulting in the development of an HR that limits pathogen spread [97,116] or that provides extreme resistance (ER) to a broad range of potyviruses [97,117,118]. The major class of *R* genes encode proteins consisting of a nucleotide-binding site (NBS), a leucine-rich repeat (LRR) region at the C-terminal, and Toll/Interleukin-1 receptor homology or a coiled-coil (CC)-domain at the N-terminal end (Figure 2B) [116].

Dominant resistance genes such as *Pvr4*, *Pvr7*, and *Pvr9* confer HR against potyviruses in pepper [21,103,105]. The *Pvr7* gene from *C. chinense* PI159236 and the *Pvr4* gene from *C. annuum* ‘CM334′ confer ER to PVY and PepMoV. *Pvr7* was tentatively re-designated as *Pvr4* in recent study [119].

*Pvr4* encodes a coiled-coil nucleotide-binding leucine-rich repeats (CNLs)-type protein, and ectopic expression of *Pvr4* in *N. benthamiana* confers resistance against PepMoV [120]. Kim et al. found that *pvr4* in the susceptible allele from *C. annuum* ‘ECW’ had higher similarity with the coiled-coil nucleotide-binding domain than with the LRR domain, which might be involved in specific recognition of Avr factors [121,122]. Researchers have demonstrated that the NIb of several potyviruses including PepMoV serves as an avirulence factor for *Pvr4* in pepper [20]. The NIb of PepMoV, pepper severe mosaic virus, and PVY induced an HR, but the NIb of TEV could not induce HR-like cell death in *Pvr4*-bearing pepper [20]. Kim et al. suggested that the differences in resistant responses among four potiviruses might be related to the low sequence identity of NIb with TEV compared with that of other potyviruses [20].

Another *R* gene, *Pvr9*, is orthologous to *Rpi-blb2* of *Solanum bulbocastanum* and was isolated via screening of Agrobacterium-based transient expression of candidate *R* genes that were able to induce an HR upon PepMoV infection in *N. benthamiana* [102]. *Pvr9* is expected to be located on pepper chromosome 6, and encodes 1298 amino acids that contain CNLs-type protein domains [21]. PepMoV infection in pepper resulted in a minor increase in *Pvr9* gene expression in the resistant cultivar *C. annuum* ‘CM334′ but in a slightly reduced expression of the susceptible allele in the susceptible cultivars *C. annuum* ‘FloralGem’ [21]. Tran et al. also demonstrated that PepMoV NIb elicits the *Pvr9*-mediated HR, which is similar to the *Pvr4*-mediated HR [20,21].

## 6. Characterization of Interacting Virus and Host Factors

### 6.1. Host Responses upon PepMoV Infection

Previous research has demonstrated that virus infection of plants affects host gene expression and metabolism, which results in altered host development and growth defects [123]. The changes in host gene/protein expression depend on whether the interaction is compatible or incompatible but also varies with plant species [123]. In early interactions between potato and PVY, for example, comparative transcriptomic analysis showed that transcriptional changes in compatible and incompatible reactions in one host shared more overall similarities in the response to PVY inoculation than compatible reactions between two different hosts [124]. The latter study also showed that a different cascade of molecular changes was triggered by two different PVY strains [124]. Although these previous studies documented changes in global gene/protein expression and in pathways in diverse host species following infection by different viruses, little is known about host responses to PepMoV infection under different conditions at a genome-wide level.

To identify pathways related to the *Pvr9*-mediated HR against PepMoV infection, researchers silenced selected genes using tobacco rattle virus-based virus-induced gene silencing and thereby assessed their functions [125]. The results showed that *Pvr9*-mediated HR requires the host genes *HSP90*, *SGT1*, *NDR1*, and *NPR1* genes but not the *EDS1* gene [125]. This indicated that *Pvr9*-mediated HR might involve the salicylic acid (SA) pathway but not the jasmonic acid (JA), ethylene (ET), reactive oxygen species (ROS), or nitric oxide (NO) pathways (Figure 1) [125]. Further research is needed to clarify the role of the SA pathway in *Pvr9*-mediated HR and the contribution of the HSP90-SGT1 complex to plant immunity against PepMoV.

Recent research showed that potato virus X vector-mediated expression of PepMoV NIa, which is highly conserved among potyviruses, resulted in severe mosaic symptoms and triggered a HR [88]. In the latter study, Gong et al. observed significantly increased expression levels of host genes including the ER-localized binding protein (Bip) and heat shock protein 90-2 (HSP90-2) in NIa-expressed plant, whereas the expression of the basic leucine zipper protein 60 (bZIP60) was not changed by NIa expression [88]. Given that Bip and HSP90-2 are required for the stabilization of many proteins in response to endoplasmic reticulum (ER) stress, the authors suggested that NIa might induce ER stress [88].

### 6.2. PepMoV–Host Interaction: Avirulence and Virulence Genes

Although the exact mechanism by which eIF4E mutations control resistance remains to be elucidated, protein–protein interaction(s) between viral elicitor(s) and the host receptor(s) might contribute to the resistance responses. In this regard, it is noteworthy that the potyviral protein VPg, which is required for viral infection, interacts with eIF4E to induce infection; mutations in eIF4E. However, prevent VPg binding and thus inhibit viral infection, resulting in a resistance response [80,115]. At the same time, amino acid substitutions in VPg that restore its binding to the mutated eIF4E can break down the resistance [18,47]. Continuous co-evolution between viral effectors and their host counterparts has apparently resulted in the diversification of both genes.

Mutation of eIF4E affects the infectivity of PepMoV in tomato [22], but there is no biological evidence for a correlation among mutated eIF4E and viral proteins of PepMoV. In the case of PVA, VPg and HC-Pro interact with each other and with eIF4E and eIF(iso)4E proteins [18,55]. A recent study revealed that HC-Pro and VPg can both interact through the eIF4E-binding motif YXXXXLΦ, which is similar to the motif in eIF4G [18]. In the latter study, Ala-Poikela et al. analyzed and compared the central region of VPg that contains a putative 4E-binding motif among 40 potyviruses [18]; they found a putative eIF4E-binding motif in the VPg of PepMoV (YADIVDV), but that motif is slightly different from that of PVA (YTDIRLI), which is similar to the eIF4E-binding motif in the VPg of PVY (YADIRDI) [8].

Several potyvirus proteins have also been identified as elicitors of resistance or determinants of avirulence, and these correspond to dominant resistance proteins in plants [126]. As previously noted, the NIb of PepMoV serves as an avirulence factor for *Pvr4* in pepper plants [20]. However, an interaction between *Pvr9* and its elicitor or so-called avirulence factor NIb of PepMoV was not detected in the model plant *N. benthamiana* [21]. The authors of the latter study suggested that the interaction depended on a third unknown factor that was present in *N. benthamiana* but not in pepper.

An HR was also triggered when *Pvr9* was co-expressed with NIbs from PepMoV, PVY, PVA, and turnip mosaic virus, but not with NIbs from zucchini yellow mosaic virus or soybean mosaic virus [21]. Although evidence was lacking for the direct binding between *Pvr9* and NIb in yeast or in plants, the mutational analyses suggested their possible relationship between *Pvr9* and NIb [21]. The amino acid substitutions E492G, V701E, F1117S, and R1160K in *Pvr9* failed to trigger an NIb-elicited HR in plants, while internal regions of NIb (the residues 186–235 and 370–445) are essential for NIb elicitor activity [21].

### 6.3. PepMoV–Host Interaction: Viral RNA Silencing Suppressors

In many potyviruses, VPg and especially HC-Pro help block or interfere with RNA silencing [47]. A recent study showed that treatment with dsRNA targeting HC-Pro or NIb inhibited PepMoV accumulation in *N. benthamiana* [127]. However, it was not clear whether this inhibitory effect was caused by reducing expression of these target genes.

As noted earlier, the use of a potato virus X-based NIa expressing vector indicated that PepMoV NIa might be responsible for symptom development in *N. benthamiana* [88]. In addition, Gong et al. found that PepMoV NIa functions as a potent suppressor of host transcriptional gene silencing by negatively affecting the DNA methylation pathway in plant hosts [88]. These results suggested that PepMoV NIa might inhibit global DNA methylation by regulating expression of essential genes involved in RNA-directed DNA methylation including *NbAGO4*, *NbMET1*, *NbDRM2*, and NbCMT3 [88]

## 7. Genome-Wide Approaches for Identifying Additional Host Factors

In the last decade, many researchers studied plant–virus interactions by focusing on genome-wide expression patterns of host and virus genes [128]. The genome-wide analyses, especially of transcriptomic data, have allowed researchers to predict some of the major biological processes that are affected by virus infection and to detect genes that are differentially expressed under specific conditions or during different stages of virus–plant interactions [128]. In *C. annuum* ‘Zunla-1′ pepper plants, for example, transcript profiles of CMV-Fny infected leaves showed different expression patterns at different time points [129]. Kim et al. reported the comprehensive transcriptomic profiling obtained from *C. annuum* at different time points after infection by *Phytophthora infestans*, PepMoV, or tobacco mosaic virus [130]. Further detailed analysis based on global transcriptomic data will be useful for identifying host factors involved in infection or resistance to infection and for elucidating host molecular networks that respond to virus infection.

## 8. Concluding Remarks and Future Prospects

PepMoV is one of the most important pathogens of solanaceous vegetables worldwide. Although the roles of each encoded PepMoV gene can be inferred by comparison with analogous genes in other potyviruses, the role(s) or function(s) of each PepMoV protein remain poorly characterized. Use of a PepMoV infectious clone will help researchers to identify the determinants of PepMoV pathogenicity/virulence and to understand the replication and movement of the virus in infected host plants.

## Figures and Tables

**Table 1:** Reported resistance genes against PepMoV.

Resistance Genes	Resistance Type	Target Virus ^a^	Source	References
*pvr1*	Recessive	PepMoV, TEV and PVY	*Capsicum chinense* PI159236 and PI152225	[22,103]
*pvr3*	Recessive	PepMoV and TEV	*C. annuum* ‘Avelar’	[104]
*Pvr4*	Dominant	PepMoV and PVY	*C. annuum* ‘CM334′	[105]
*Pvr7*	Dominant	PepMoV	*C. chinense* PI159236	[103]
*Pvr9*	Dominant	PepMoV	*C. annuum* ‘CM334′	[21]

^a^ PepMoV, pepper mottle virus; TEV, tobacco etch virus; PVY, potato virus Y.

## Data Availability

The data presented in this study are available in the article.

## References

[1] Valli A., García J.A., López-Moya J.J. (2015). Potyviridae. eLS.

[2] Zitter T.A. (1972). Plant disease reporter naturally occurring pepper virus strains in south Florida. Plant Dis. Rep..

[3] Ogawa Y., Hagiwara K., Iwai H., Izumi S., Arai K. (2003). First report of pepper mottle virus on *Capsicum annuum* in Japan. J. Gen. Plant Pathol..

[4] Kim Y.-J., Jonson M.G., Choi H.S., Ko S.-J., Kim K.-H. (2009). Molecular characterization of Korean pepper mottle virus isolates and its relationship to symptom variations. Virus Res..

[5] Rodriguez-Alvarado G., Fernandez-Pavia S., Creamer R., Liddell C. (2002). *Pepper mottle virus* causing disease in chile peppers in southern New Mexico. Plant Dis..

[6] Verhoeven J.T.J., Willemen T., Roenhorst J. (2002). First report of pepper mottle virus in tomato. Plant Dis..

[7] Melzer M., Sugano J., Cabanas D., Dey K., Kandouh B., Mauro D., Rushanaedy I., Srivastava S., Watanabe S., Borth W. (2012). First report of pepper mottle virus infecting tomato in Hawaii. Plant Dis..

[8] Cheng Y., Deng T., Chen C., Liao J., Chang C.-A., Chiang C. (2011). First report of pepper mottle virus in bell pepper in Taiwan. Plant Dis..

[9] Kaur S., Kang S., Sharma A., Sharma S. (2014). First report of pepper mottle virus infecting chilli pepper in India. New Dis. Rep..

[10] Tangjang S., Reddy M.S., Suryanarayanan T.S., Taka T. (2018). Seed transmissibility of pepper mottle virus: Survival of virus. Curr. Sci..

[11] Zitter T.A. (1975). Transmission of pepper mottle virus from susceptible and resistant pepper cultivars. Phytopathology.

[12] Han J.-H., Choi H.-S., Kim D.-H., Lee H.-R., Kim B.-D. (2006). Biological, physical and cytological properties of pepper mottle virus-SNU1 and its RT-PCR detection. Plant Pathol. J..

[13] Kim M.-K., Kwak H.-R., Han J.-H., Ko S.-J., Lee S.-H., Park J.-W., Jonson M.G., Kim K.-H., Kim J.-S., Choi H.-S. (2008). Isolation and characterization of pepper mottle virus infecting tomato in Korea. Plant Pathol. J..

[14] Murphy J.F., Bowen K.L. (2006). Synergistic disease in pepper caused by the mixed infection of cucumber mosaic virus and pepper mottle virus. Phytopathology.

[15] Murphy J.F., Kyle M.M. (1995). Alleviation of restricted systemic spread of pepper mottle potyvirus in *Capsicum annuum* cv. Avelar by coinfection with a cucumovirus. Phytopathology.

[16] Warren C.E., Murphy J. (2003). The complete nucleotide sequence of pepper mottle virus-Florida RNA. Arch. Virol..

[17] Vance V.B., Moore D., Turpen T.H., Bracker A., Hollowell V.C. (1992). The complete nucleotide sequence of pepper mottle virus genomic RNA: Comparison of the encoded polyprotein with those of other sequenced potyviruses. Virology.

[18] Ala-Poikela M., Rajamäki M.-L., Valkonen J. (2019). A novel interaction network used by potyviruses in virus–host interactions at the protein Level. Viruses.

[19] Jonson M.G., Seo J.-K., Cho H.-S., Kim J.-S., Kim K.-H. (2009). Effects of recombination on the pathogenicity and evolution of pepper mottle virus. Plant Pathol. J..

[20] Kim S.-B., Lee H.-Y., Seo S., Lee J.H., Choi D. (2015). RNA-dependent RNA polymerase (NIb) of the potyviruses is an avirulence factor for the broad-spectrum resistance gene *Pvr4* in *Capsicum annuum* cv. CM334. PLoS ONE.

[21] Tran P.-T., Choi H., Choi D., Kim K.-H. (2015). Molecular characterization of *Pvr9* that confers a hypersensitive response to pepper mottle virus (a potyvirus) in *Nicotiana benthamiana*. Virology.

[22] Yoon Y.-J., Venkatesh J., Lee J.-H., Kim J., Lee H.-E., Kim D.-S., Kang B.-C. (2020). Genome editing of eIF4E1 in tomato confers resistance to pepper mottle virus. Front. Plant Sci..

[23] Tran P.-T., Fang M., Widyasari K., Kim K.-H. (2019). A plant intron enhances the performance of an infectious clone in planta. J. Virol. Methods.

[24] Song E.G., Ryu K.H. (2017). A pepper mottle virus-based vector enables systemic expression of endoglucanase D in non-transgenic plants. Arch. Virol..

[25] Gibbs A.J., Hajizadeh M., Ohshima K., Jones R.A. (2020). The potyviruses: An evolutionary synthesis is emerging. Viruses.

[26] Wylie S.J., Adams M., Chalam C., Kreuze J., López-Moya J.J., Ohshima K., Praveen S., Rabenstein F., Stenger D., Wang A. (2017). ICTV virus taxonomy profile: *Potyviridae*. J. Gen. Virol..

[27] Purcifull D., Zitter T.A., Hiebert E. (1975). Morphology, host range and serological relationships of pepper mottle virus. Phytopathology.

[28] Hiebert E., Purcifull D. (1992). A comparison of pepper mottle virus with potato virus Y and evidence for their distinction. Potyvirus Taxonomy.

[29] Lee M.Y., Song Y.S., Ryu K.H. (2011). Development of infectious transcripts from full-length and GFP-tagged cDNA clones of *Pepper mottle virus* and stable systemic expression of GFP in tobacco and pepper. Virus Res..

[30] Zhang Y., Luo X., Zhang D., OuYang X., Zhang Z., Li F., Zhang C., Chen J., Zhou X., Zhang S. (2019). Genome and phylogenetic analyses of chinese pepper mottle virus isolates from chili pepper plants. J. Plant Pathol..

[31] Revers F., García J.A. (2015). Molecular biology of potyviruses. Adv. Virus Res..

[32] Chung B.Y.-W., Miller W.A., Atkins J.F., Firth A.E. (2008). An overlapping essential gene in the *Potyviridae*. Proc. Natl. Acad. Sci. USA.

[33] Mäkinen K., Hafrén A. (2014). Intracellular coordination of potyviral RNA functions in infection. Front. Plant Sci..

[34] Urcuqui-Inchima S., Haenni A.-L., Bernardi F. (2001). Potyvirus proteins: A wealth of functions. Virus Res..

[35] Satyanarayana T., Gowda S., Ayllón M.A., Dawson W. (2003). Frameshift mutations in infectious cDNA clones of citrus tristeza virus: A strategy to minimize the toxicity of viral sequences to *Escherichia coli*. Virology.

[36] Bedoya L.C., Daròs J.-A. (2010). Stability of tobacco etch virus infectious clones in plasmid vectors. Virus Res..

[37] Gao Q., Chou Y.-Y., Doğanay S., Vafabakhsh R., Ha T., Palese P. (2012). The influenza A virus PB2, PA, NP, and M segments play a pivotal role during genome packaging. J. Virol..

[38] Johansen I.E. (1996). Intron insertion facilitates amplification of cloned virus cDNA in Escherichia coli while biological activity is reestablished after transcription in vivo. Proc. Natl. Acad. Sci. USA.

[39] Olsen B., Johansen I.E. (2001). Nucleotide sequence and infectious cDNA clone of the L1 isolate of pea seed-borne mosaic potyvirus. Arch. Virol..

[40] López-Moya J.J., García J.A. (2000). Construction of a stable and highly infectious intron-containing cDNA clone of plum pox potyvirus and its use to infect plants by particle bombardment. Virus Res..

[41] Tuo D., Shen W., Yan P., Li X., Zhou P. (2015). Rapid construction of stable infectious full-length cDNA clone of papaya leaf distortion mosaic virus using in-fusion cloning. Viruses.

[42] Constantin G.D., Krath B.N., MacFarlane S.A., Nicolaisen M., Johansen I.E., Lund O.S. (2004). Virus-induced gene silencing as a tool for functional genomics in a legume species. Plant J..

[43] Ratcliff F., Martin-Hernandez A.M., Baulcombe D.C. (2001). Technical advance: Tobacco rattle virus as a vector for analysis of gene function by silencing. Plant J..

[44] Deng P., Wu Z., Wang A. (2015). The multifunctional protein CI of potyviruses plays interlinked and distinct roles in viral genome replication and intercellular movement. Virol. J..

[45] Dolja V.V., McBride H.J., Carrington J.C. (1992). Tagging of plant potyvirus replication and movement by insertion of beta-glucuronidase into the viral polyprotein. Proc. Natl. Acad. Sci. USA.

[46] Quenouille J., Vassilakos N., Moury B. (2013). Potato virus Y: A major crop pathogen that has provided major insights into the evolution of viral pathogenicity. Mol. Plant Pathol..

[47] Yang X., Li Y., Wang A. (2021). Research advances in potyviruses: From the laboratory bench to the field. Annu. Rev. Phytopathol..

[48] Verchot J., Carrington J.C. (1995). Evidence that the potyvirus P1 proteinase functions in trans as an accessory factor for genome amplification. J. Virol..

[49] Nakahara K.S., Shimada R., Choi S.-H., Yamamoto H., Shao J., Uyeda I. (2010). Involvement of the P1 cistron in overcoming eIF4E-mediated recessive resistance against clover yellow vein virus in pea. Mol. Plant-Microbe Interact..

[50] Pasin F., Simón-Mateo C., García J.A. (2014). The hypervariable amino-terminus of P1 protease modulates potyviral replication and host defense responses. PLoS Pathog..

[51] Maia I.G., Haenni A.-L., Bernardi F. (1996). Potyviral HC-Pro: A multifunctional protein. J. Gen. Virol..

[52] Hasiów-Jaroszewska B., Fares M.A., Elena S.F. (2014). Molecular evolution of viral multifunctional proteins: The case of potyvirus HC-Pro. J. Mol. Evol..

[53] Blanc S., Ammar E.D., Garcia-Lampasona S., Dolja V.V., Llave C., Baker J., Pirone T.P. (1998). Mutations in the potyvirus helper component protein: Effects on interactions with virions and aphid stylets. J. Gen. Virol..

[54] Jin Y., Ma D., Dong J., Jin J., Li D., Deng C., Wang T. (2007). HC-Pro protein of potato virus Y can interact with three *Arabidopsis* 20S proteasome subunits in planta. J. Virol..

[55] Ala-Poikela M., Goytia E., Haikonen T., Rajamäki M.-L., Valkonen J.P. (2011). Helper component proteinase of the genus *Potyvirus* is an interaction partner of translation initiation factors eIF (iso) 4E and eIF4E and contains a 4E binding motif. J. Virol..

[56] Langenberg W.G., Zhang L. (1997). Immunocytology shows the presence of tobacco etch virus P3 protein in nuclear inclusions. J. Struct. Biol..

[57] Cui X., Wei T., Chowda-Reddy R.V., Sun G., Wang A. (2010). The tobacco etch virus P3 protein forms mobile inclusions via the early secretory pathway and traffics along actin microfilaments. Virology.

[58] Wei T., Zhang C., Hong J., Xiong R., Kasschau K.D., Zhou X., Carrington J.C., Wang A. (2010). Formation of complexes at plasmodesmata for potyvirus intercellular movement is mediated by the viral protein P3N-PIPO. PLoS Pathog..

[59] Jenner C.E., Wang X., Tomimura K., Ohshima K., Ponz F., Walsh J.A. (2003). The dual role of the potyvirus P3 protein of turnip mosaic virus as a symptom and avirulence determinant in brassicas. Mol. Plant-Microbe Interact..

[60] Cui H., Wang A. (2016). Plum pox virus 6K1 protein is required for viral replication and targets the viral replication complex at the early stage of infection. J. Virol..

[61] Sorel M., Garcia J.A., German-Retana S. (2014). The *Potyviridae* cylindrical inclusion helicase: A key multipartner and multifunctional protein. Mol. Plant-Microbe Interact..

[62] Carrington J.C., Jensen P.E., Schaad M.C. (1998). Genetic evidence for an essential role for potyvirus CI protein in cell-to-cell movement. Plant J..

[63] Tavert-Roudet G., Abdul-Razzak A., Doublet B., Walter J., Delaunay T., German-Retana S., Michon T., Le Gall O., Candresse T. (2012). The C terminus of lettuce mosaic potyvirus cylindrical inclusion helicase interacts with the viral VPg and with lettuce translation eukaryotic initiation factor 4E. J. Gen. Virol..

[64] Abdul-Razzak A., Guiraud T., Peypelut M., Walter J., Houvenaghel M.C., Candresse T., Le Gall O., German-Retana S. (2009). Involvement of the cylindrical inclusion (CI) protein in the overcoming of an eIF4E-mediated resistance against lettuce mosaic potyvirus. Mol. Plant Pathol..

[65] Sorel M., Svanella-Dumas L., Candresse T., Acelin G., Pitarch A., Houvenaghel M.-C., German-Retana S. (2014). Key mutations in the cylindrical inclusion involved in Lettuce mosaic virus adaptation to eIF4E-mediated resistance in lettuce. Mol. Plant-Microbe Interact..

[66] Spetz C., Valkonen J.P. (2004). Potyviral 6K2 protein long-distance movement and symptom-induction functions are independent and host-specific. Mol. Plant-Microbe Interact..

[67] Jiang J., Patarroyo C., Cabanillas D.G., Zheng H., Laliberté J.-F. (2015). The vesicle-forming 6K2 protein of turnip mosaic virus interacts with the COPII coatomer Sec24a for viral systemic infection. J. Virol..

[68] González R., Wu B., Li X., Martínez F., Elena S.F. (2019). Mutagenesis scanning uncovers evolutionary constraints on tobacco etch potyvirus membrane-associated 6K2 protein. Genome Biol. Evol..

[69] Shen W., Shi Y., Dai Z., Wang A. (2020). The RNA-dependent RNA polymerase NIb of potyviruses plays multifunctional, contrasting roles during viral infection. Viruses.

[70] Wei T., Wang A. (2008). Biogenesis of cytoplasmic membranous vesicles for plant potyvirus replication occurs at endoplasmic reticulum exit sites in a COPI-and COPII-dependent manner. J. Virol..

[71] Restrepo-Hartwig M.A., Carrington J.C. (1994). The tobacco etch potyvirus 6-kilodalton protein is membrane associated and involved in viral replication. J. Virol..

[72] Lõhmus A., Varjosalo M., Mäkinen K. (2016). Protein composition of 6K2-induced membrane structures formed during potato virus A infection. Mol. Plant Pathol..

[73] Lerich A., Langhans M., Sturm S., Robinson D.G. (2011). Is the 6 kDa tobacco etch viral protein a bona fide ERES marker?. J. Exp. Bot..

[74] Jiang J., Laliberté J.-F. (2011). The genome-linked protein VPg of plant viruses—a protein with many partners. Curr Opin Virol..

[75] Eskelin K., Hafrén A., Rantalainen K.I., Mäkinen K. (2011). Potyviral VPg enhances viral RNA translation and inhibits reporter mRNA translation in planta. J. Virol..

[76] de Oliveira L.C., Volpon L., Rahardjo A.K., Osborne M.J., Culjkovic-Kraljacic B., Trahan C., Oeffinger M., Kwok B.H., Borden K.L. (2019). Structural studies of the eIF4E–VPg complex reveal a direct competition for capped RNA: Implications for translation. Proc. Natl. Acad. Sci. USA.

[77] Khan M.A., Miyoshi H., Gallie D.R., Goss D.J. (2008). Potyvirus genome-linked protein, VPg, directly affects wheat germ in vitro translation: Interactions with translation initiation factors eIF4F and eIFiso4F. J. Biol. Chem..

[78] Grzela R., Strokovska L., Andrieu J.-P., Dublet B., Zagorski W., Chroboczek J. (2006). Potyvirus terminal protein VPg, effector of host eukaryotic initiation factor eIF4E. Biochimie.

[79] Miyoshi H., Okade H., Muto S., Suehiro N., Nakashima H., Tomoo K., Natsuaki T. (2008). Turnip mosaic virus VPg interacts with Arabidopsis thaliana eIF (iso) 4E and inhibits in vitro translation. Biochimie.

[80] Wang A., Krishnaswamy S. (2012). Eukaryotic translation initiation factor 4E-mediated recessive resistance to plant viruses and its utility in crop improvement. Mol. Plant Pathol..

[81] Kang B.-C., Yeam I., Jahn M.M. (2005). Genetics of plant virus resistance. Annu. Rev. Phytopathol..

[82] Dufresne P.J., Thivierge K., Cotton S., Beauchemin C., Ide C., Ubalijoro E., Laliberté J.-F., Fortin M.G. (2008). Heat shock 70 protein interaction with turnip mosaic virus RNA-dependent RNA polymerase within virus-induced membrane vesicles. Virology.

[83] Cheng X., Wang A. (2017). The potyvirus silencing suppressor protein VPg mediates degradation of SGS3 via ubiquitination and autophagy pathways. J. Virol..

[84] Rodamilans B., Shan H., Pasin F., García J.A. (2018). Plant viral proteases: Beyond the role of peptide cutters. Front. Plant Sci..

[85] Mestre P., Brigneti G., Baulcombe D.C. (2000). An Ry-mediated resistance response in potato requires the intact active site of the NIa proteinase from potato virus Y. Plant J..

[86] Casteel C.L., De Alwis M., Bak A., Dong H., Whitham S.A., Jander G. (2015). Disruption of ethylene responses by turnip mosaic virus mediates suppression of plant defense against the green peach aphid vector. Plant Physiol..

[87] Bak A., Cheung A.L., Yang C., Whitham S.A., Casteel C.L. (2017). A viral protease relocalizes in the presence of the vector to promote vector performance. Nat. Commun..

[88] Gong Y.-N., Tang R.-Q., Zhang Y., Peng J., Xian O., Zhang Z.-H., Zhang S.-B., Zhang D.-Y., Liu H., Luo X.-W. (2020). The NIa-protease protein encoded by the *Pepper mottle virus* is a pathogenicity determinant and releases DNA methylation of *Nicotiana benthamiana*. Front. Microbiol..

[89] Kežar A., Kavčič L., Polák M., Nováček J., Gutiérrez-Aguirre I., Žnidarič M.T., Coll A., Stare K., Gruden K., Ravnikar M. (2019). Structural basis for the multitasking nature of the potato virus Y coat protein. Sci. Adv..

[90] Martínez-Turiño S., García J.A. (2020). Potyviral coat protein and genomic RNA: A striking partnership leading virion assembly and more. Adv. Virus Res..

[91] Dolja V.V., Haldeman-Cahill R., Montgomery A.E., Vandenbosch K.A., Carrington J.C. (1995). Capsid protein determinants involved in cell-to-cell and long distance movement of tobacco etch potyvirus. Virology.

[92] Besong-Ndika J., Ivanov K.I., Hafrèn A., Michon T., Mäkinen K. (2015). Cotranslational coat protein-mediated inhibition of potyviral RNA translation. J. Virol..

[93] Dai Z., He R., Bernards M.A., Wang A. (2020). The *cis*-expression of the coat protein of turnip mosaic virus is essential for viral intercellular movement in plants. Mol. Plant Pathol..

[94] Arazi T., Shiboleth Y., Gal-On A. (2001). A nonviral peptide can replace the entire N terminus of zucchini yellow mosaic potyvirus coat protein and permits viral systemic infection. J. Virol..

[95] Dolja V., Haldeman R., Robertson N., Dougherty W., Carrington J. (1994). Distinct functions of capsid protein in assembly and movement of tobacco etch potyvirus in plants. EMBO J..

[96] Yan Z.-Y., Cheng D.-J., Liu L.-Z., Geng C., Tian Y.-P., Li X.-D., Valkonen J.P.T. (2021). The conserved aromatic residue W122 is a determinant of potyviral coat protein stability, replication, and cell-to-cell movement in plants. Mol. Plant Pathol..

[97] Baebler Š., Coll A., Gruden K. (2020). Plant molecular responses to Potato Virus Y: A continuum of outcomes from sensitivity and tolerance to resistance. Viruses.

[98] Seo J.K., Kang S.H., Seo B.Y., Jung J.K., Kim K.-H. (2010). Mutational analysis of interaction between coat protein and helper component-proteinase of Soybean mosaic virus involved in aphid transmission. Mol. Plant Pathol..

[99] Llave C., Martinez B., Diaz-Ruiz J., Lopez-Abella D. (2002). Amino acid substitutions within the Cys-rich domain of the tobacco etch potyvirus HC-Pro result in loss of transmissibility by aphids. Arch. Virol..

[100] Mandadi K.K., Scholthof K.-B.G. (2013). Plant immune responses against viruses: How does a virus cause disease?. Plant Cell.

[101] Li F., Wang A. (2019). RNA-targeted antiviral immunity: More than just RNA silencing. Trends Microbiol..

[102] Tran P.-T., Choi H., Kim S.-B., Lee H.-A., Choi D., Kim K.-H. (2014). A simple method for screening of plant NBS-LRR genes that confer a hypersensitive response to plant viruses and its application for screening candidate pepper genes against *Pepper mottle virus*. J. Virol. Methods.

[103] Liu L., Venkatesh J., Jo Y.D., Koeda S., Hosokawa M., Kang J.-H., Goritschnig S., Kang B.-C. (2016). Fine mapping and identification of candidate genes for the *sy-2* locus in a temperature-sensitive chili pepper (*Capsicum chinense*). Theor. Appl. Genet..

[104] Murphy J.F., Blauth J.R., Livingstone K.D., Lackney V.K., Jahn M.K. (1998). Genetic mapping of the *pvr1* locus in *Capsicum* spp. and evidence that distinct potyvirus resistance loci control responses that differ at the whole plant and cellular levels. Mol. Plant-Microbe Interact..

[105] Janzac B., Fabre M.F., Palloix A., Moury B. (2009). Phenotype and spectrum of action of the *Pvr4* resistance in pepper against potyviruses, and selection for virulent variants. Plant Pathol..

[106] Robaglia C., Caranta C. (2006). Translation initiation factors: A weak link in plant RNA virus infection. Trends Plant Sci..

[107] Kyle M., Palloix A. (1997). Proposed revision of nomenclature for potyvirusresistance genes in *Capsicum*. Euphytica.

[108] Pavan S., Jacobsen E., Visser R.G., Bai Y. (2010). Loss of susceptibility as a novel breeding strategy for durable and broad-spectrum resistance. Mol. Breed..

[109] Guerini M.N., Murphy J.F. (1999). Resistance of *Capsicum annuum* ‘Avelar’to pepper mottle potyvirus and alleviation of this resistance by co-infection with cucumber mosaic cucumovirus are associated with virus movement. J. Gen. Virol..

[110] Ruffel S., Gallois J.-L., Moury B., Robaglia C., Palloix A., Caranta C. (2006). Simultaneous mutations in translation initiation factors eIF4E and eIF(iso)4E are required to prevent pepper veinal mottle virus infection of pepper. J. Gen. Virol..

[111] Yeam I., Kang B.-C., Lindeman W., Frantz J.D., Faber N., Jahn M.M. (2005). Allele-specific CAPS markers based on point mutations in resistance alleles at the *pvr1* locus encoding eIF4E in *Capsicum*. Theor. Appl. Genet..

[112] Ruffel S., Gallois J.-L., Lesage M., Caranta C. (2005). The recessive potyvirus resistance gene *pot-1* is the tomato orthologue of the pepper *pvr2-eIF4E* gene. Mol. Genet. Genom..

[113] Kang B.C., Yeam I., Li H., Perez K.W., Jahn M.M. (2007). Ectopic expression of a recessive resistance gene generates dominant potyvirus resistance in plants. Plant Biotechnol. J..

[114] Gauffier C., Lebaron C., Moretti A., Constant C., Moquet F., Bonnet G., Caranta C., Gallois J.L. (2016). A TILLING approach to generate broad-spectrum resistance to potyviruses in tomato is hampered by eIF4E gene redundancy. Plant J..

[115] Kang B.C., Yeam I., Frantz J.D., Murphy J.F., Jahn M.M. (2005). The *pvr1* locus in *Capsicum* encodes a translation initiation factor eIF4E that interacts with tobacco etch virus VPg. Plant J..

[116] de Ronde D., Butterbach P., Kormelink R. (2014). Dominant resistance against plant viruses. Front. Plant Sci..

[117] Collmer C.W., Marston M.F., Taylor J.C., Jahn M. (2000). The I gene of bean: A dosage-dependent allele conferring extreme resistance, hypersensitive resistance, or spreading vascular necrosis in response to the potyvirus bean common mosaic virus. Mol. Plant-Microbe Interact..

[118] Zhang C., Grosic S., Whitham S.A., Hill J.H. (2012). The requirement of multiple defense genes in soybean *Rsv1*–mediated extreme resistance to soybean mosaic virus. Mol. Plant-Microbe Interact..

[119] Venkatesh J., An J., Kang W.-H., Jahn M., Kang B.-C. (2018). Fine mapping of the dominant potyvirus resistance gene *Pvr7* reveals a relationship with *Pvr4* in *Capsicum annuum*. Phytopathology.

[120] Kim S.B., Kang W.H., Huy H.N., Yeom S.I., An J.T., Kim S., Kang M.Y., Kim H.J., Jo Y.D., Ha Y. (2017). Divergent evolution of multiple virus-resistance genes from a progenitor in *Capsicum* spp.. New Phytol..

[121] Mondragón-Palomino M., Meyers B.C., Michelmore R.W., Gaut B.S. (2002). Patterns of positive selection in the complete NBS-LRR gene family of *Arabidopsis thaliana*. Genome Res..

[122] Goritschnig S., Steinbrenner A.D., Grunwald D.J., Staskawicz B.J. (2016). Structurally distinct *Arabidopsis thaliana* NLR immune receptors recognize tandem WY domains of an oomycete effector. New Phytol..

[123] Whitham S.A., Yang C., Goodin M.M. (2006). Global impact: Elucidating plant responses to viral infection. Mol. Plant-Microbe Interact..

[124] Goyer A., Hamlin L., Crosslin J.M., Buchanan A., Chang J.H. (2015). RNA-Seq analysis of resistant and susceptible potato varieties during the early stages of potato virus Y infection. BMC Genom..

[125] Tran P.-T., Choi H., Choi D., Kim K.-H. (2016). Virus-induced gene silencing reveals signal transduction components required for the *Pvr9*-mediated hypersensitive response in *Nicotiana benthamiana*. Virology.

[126] Huang C. (2021). From player to pawn: Viral avirulence factors involved in plant immunity. Viruses.

[127] Yoon J., Fang M., Lee D., Park M., Kim K.-H., Shin C. (2021). Double-stranded RNA confers resistance to pepper mottle virus in *Nicotiana benthamiana*. Appl. Biol. Chem..

[128] Zanardo L.G., de Souza G.B., Alves M.S. (2019). Transcriptomics of plant–virus interactions: A review. Theor. Exp. Plant Physiol..

[129] Zhu C., Li X., Zheng J. (2018). Transcriptome profiling using Illumina-and SMRT-based RNA-seq of hot pepper for in-depth understanding of genes involved in CMV infection. Gene.

[130] Kim M.-S., Kim S., Jeon J., Kim K.-T., Lee H.-A., Lee H.-Y., Park J., Seo E., Kim S.-B., Yeom S.-I. (2018). Global gene expression profiling for fruit organs and pathogen infections in the pepper, *Capsicum annuum* L.. Sci. Data.

